# Metabolomics of chronic obstructive pulmonary disease and obstructive sleep apnea syndrome: response to Maniscalco and Motta

**DOI:** 10.1007/s11306-015-0945-x

**Published:** 2016-01-14

**Authors:** P. Mlynarz, S. Deja, I. Stanimirova, A. Zabek, W. Barg, R. Jankowska

**Affiliations:** Department of Bioorganic Chemistry, Wrocław University of Technology, Wybrzeze Wyspianskiego 27, 50-370 Wroclaw, Poland; Faculty of Chemistry, Opole University, Pl. Kopernika 11a, 45-040 Opole, Poland; Institute of Chemistry, The University of Silesia, Szkolna 9, 40-006 Katowice, Poland; Department of Physiology, Wroclaw Medical University, T. Chalubinskiego 10, 50-368 Wroclaw, Poland; Department and Clinic of Pulmonology and Lung Cancers, Wroclaw Medical University, Grabiszynska 105, 53-439 Wroclaw, Poland

We appreciate Maniscalco and Motta’s comments on our recently published article “Fusion of the ^1^H NMR data of serum, urine and exhaled breath condensate in order to discriminate chronic obstructive pulmonary disease and obstructive sleep apnea syndrome” (Zabek et al. 2015) and we are grateful for the opportunity to clarify a number of points from our work. We are glad that the authors appreciated our data analysis and interpretation.

Fusion of metabolomic data is an outstanding tool for a comprehensive description of patient’s phenotype that combines metabolic profiles acquired in different domains. Such an integrative approach is highly valued in the so-called ‘breathomics’ (Smolinska et al. 2014) for a better understanding of the complex respiratory diseases and discovering their biomarkers (Sterk et al. 2013). Our work was an attempt to provide a novel comprehensive description of metabolic phenotype and to test its utility in order to discriminate between two closely related respiratory pathological conditions.

We performed a very careful selection of patients and those with an overlap syndrome who suffered from both COPD and OSAS (a total of 21 individuals) were not considered in our study. A further reduction in the number of patients was obtained by eliminating patients who did not deliver samples of all three biofluids for analysis. Therefore, even though a total of 85 serum, 91 urine and 82 EBC samples were collected, only 46 individuals (18 patients with COPD and 28 patients with the OSA syndrome) were finally included in the study. The selection of samples is in agreement with the assumption that changes in the level of a metabolite or a combination of metabolites present in different biofluids may be characteristic for a disease at all stages of its progression, regardless of the degree of the severity of the disease, obesity or smoking habits of the patients. Therefore, in our opinion, the hypothesis suggested by Maniscalco and Motta that the ‘lower specificity’ of the models combining EBC and other biofluids might be related to the severity of the disease would require further verification using specifically designed data. In order to assess the effect of the severity of a disease, obesity or smoking habits on the metabolic profiles, analysis of data in which a strict categorization of COPD and OSAS patients according to their clinical profiles is required. Unfortunately, it was not possible to present an analysis of such designed data in our article due to the small number of samples. Stratification of patients based on spirometric results was out of the scope of this study. A comparison of our results and the results presented in the recent publication authored by Maniscalco and Motta (Paris et al. 2015) is hindered due to the differences in normalization methods that were used. We are currently developing a more comprehensive clinical trial that we hope will provide a clearer answer to the questions that have been raised.

It is our conviction that our work will be a useful contribution to the field of metabolomics and will help to better understand the metabolic mechanisms of respiratory pathologies.
